# Empowering health geography research with location-based social media data: innovative food word expansion and energy density prediction via word embedding and machine learning

**DOI:** 10.1186/s12942-023-00344-5

**Published:** 2023-09-16

**Authors:** Jue Wang, Gyoorie Kim, Kevin Chen-Chuan Chang

**Affiliations:** 1 Department of Geography and Planning, University of Toronto, 100 St. George Street, Toronto, ON M5S 3G3 Canada; 2 Department of Geography, Geomatics and Environment, University of Toronto Mississauga, 3359 Mississauga Road, Mississauga, ON L5L 1C6 Canada; 3 Department of Computer Science, University of Illinois at Urbana-Champaign, 201 North Goodwin Avenue, Urbana, IL USA

**Keywords:** Food environment, Food words, Food energy density, Machine learning, Health geography, Geographic information science

## Abstract

**Background:**

The exponential growth of location-based social media (LBSM) data has ushered in novel prospects for investigating the urban food environment in health geography research. However, previous studies have primarily relied on word dictionaries with a limited number of food words and employed common-sense categorizations to determine the healthiness of those words. To enhance the analysis of the urban food environment using LBSM data, it is crucial to develop a more comprehensive list of food-related words. Within the context, this study delves into the exploration of expanding food-related words along with their associated energy densities.

**Methods:**

This study addresses the aforementioned research gap by introducing a novel methodology for expanding the food-related word dictionary and predicting energy densities. Seed words are generated from official and crowdsourced food composition databases, and new food words are discovered by clustering food words within the word embedding space using the Gaussian mixture model. Machine learning models are employed to predict the energy density classifications of these food words based on their feature vectors. To ensure a thorough exploration of the prediction problem, ten widely used machine learning models are evaluated.

**Results:**

The approach successfully expands the food-related word dictionary and accurately predicts food energy density (reaching 91.62%.). Through a comparison of the newly expanded dictionary with the initial seed words and an analysis of Yelp reviews in the city of Toronto, we observe significant improvements in identifying food words and gaining a deeper understanding of the food environment.

**Conclusions:**

This study proposes a novel method to expand food-related vocabulary and predict the food energy density based on machine learning and word embedding. This method makes a valuable contribution to building a more comprehensive list of food words that can be used in geography and public health studies by mining geotagged social media data.

**Supplementary Information:**

The online version contains supplementary material available at 10.1186/s12942-023-00344-5.

## Background

As urbanization continues to rapidly progress, urban spaces are becoming increasingly complex, resulting in the emergence of heterogeneous urban environments. With the abundance of location-based social media data and the spatial information it provides, advanced studies are now possible [1] to understand the urban environment and investigate human interactions with urban spaces across geographic regions [2–6].

Traditionally, information about the urban environment was derived from authoritative datasets such as nationwide surveys (census), remote sensing imageries, light detection and ranging data, and geographic information systems (GIS). Remote sensing data have been widely used to classify and monitor urban land use and functions [7–10], while GIS data have been used to derive the urban built environment from different perspectives, such as walkability [11–13] and livability [14–16]. Social environment characteristics have also been aggregated using GIS and census data at different scales [17, 18].

However, the data used in these conventional studies are mostly generated and aggregated by governments or authorities [19]. They typically only cover the physical aspect of the urban space or simple inferences of social environment characteristics from census data, while citizens' perceptions and experiences of the urban space are mostly ignored [19]. Moreover, data collection using these methods is labour intensive and time-consuming, limiting the study to a relatively large scale and lacking fine-scale attributes. Additionally, there is always a temporal lag between when the data are collected and when they are publicly available for use.

To enrich the knowledge of the urban environment and interactions between citizens and urban spaces at a fine scale, scholars have tried to integrate the experiences and perceptions of urban spaces by local citizens. Researchers have used focus group surveys, interviews, observation of places, and cognitive mapping to understand the urban environment [19–21]. Geo-narrative is one of the widely utilized ways to derive local knowledge of urban spaces. This approach applies the semantic analysis of geospatial-related narrative content, such as travel logs, oral histories and biographies [22], to help bridge the semantic gap between human's perception of space and the urban environment [23]. For example, researchers have employed geo-narrative to investigate the citizens’ perception and experience of the green and blue space in their daily life [24], to explore qualitative activity space according to individual perception of the urban environment [25], to infer thematic places based on individual sense of place [26].

Although the advancements in geo-narrative techniques improved the capability to process geospatial-related narrative materials and advanced the understanding of urban spaces based on local knowledge, they have been constrained by the quantity and quality of available data sources [27]. However, the popularity of social media platforms, such as Twitter, Instagram, Facebook, and Foursquare, has led to more people using these mediums to share their thoughts and opinions. These billions of posts generated worldwide can be served as a rich source of information about personal experiences and public perspectives. Most social media platforms allow the user to track their location (geographic coordinates) embedded in their posts, namely Location-based Social Media (LBSM). The pervasiveness of LBSM provides large volumes of spatial data combined with sociodemographic information generated by real-world users in real-time [28]. Unlike conventional ways of data collection, multiple fields have benefited from mining this rapidly available data to profile different environments, including political analyses [29–32], natural hazards and disaster management [33–35], epidemiology outbreak tracking [36–39], medical and pharmaceutical services [40, 41], and others [42–44].

A growing trend in urban environment analyses is the use of publicly accessible LBSM data sources [3, 4, 45]. Within LBSM data, users provide real-time information about urban environments [46, 47] twenty-four hours a day, seven days a week. Although the LBSM data are often being criticized for their biased representation of the population (e.g., the users tend to be younger) and noise [48], utilizing social media data as a supplementary data source for urban environment studies has value since the information on millions of users' opinions and daily behaviours can enrich the conventional GIS data sources [49, 50] if tuned to the right frequency (like radio waves in the air).

With the rapid advancements in machine learning (ML) and natural language processing (NLP) techniques, there is a growing trend towards harnessing these technologies to analyze urban environment through the lens of human perception utilizing geotagged social media data [51–54]. NLP proves invaluable in exploring unstructured text data [55], such as social media posts, where the integration of sentiment analysis through machine learning, encompassing techniques like artificial neural network and support vector machine, can unveil hidden patterns and emerging trends within vast social media datasets [56, 57]. By incorporating the location details from the geotags associated with social media data, the synergistic utilization of ML and NLP offers a potent means to comprehensively understand the various facets of the urban environment as perceived by individuals [58]. Illustratively, this synergy can pinpoint tourist hotspots using Flickr imagery and travel blogs [26, 59], unveil citizens' conceptualization of places via geo-referenced tweets or micro lifelogs [47, 60], extract urban functional regions from tweets, Foursquare venues, and user check-in behaviour [45, 61], and analyze the urban food environment through geotagged tweets [62, 63].

Among the different urban environments, the food environment—the environment within which we make our daily food choices—is a critical factor contributing to obesity [64]. According to the Health Canada [65], obesity in adults is defined as having a body mass index (BMI) of 30 or greater, while overweight is defined as a BMI of 25 to less than 30. The prevalence of obesity in North America has rapidly increased [66]. In March 2020, the U.S. obesity prevalence increased to 41.9% [67]. In Canada, about 35% of adults have either overweight or obese as of 2021 [68]. Evidence shows that exposure to an unhealthy food environment (e.g., high density of fast food outlets) has significant associations with obesity and other obesity-related chronic diseases [69, 70].

In spatially focused food environment studies, researchers are increasingly turning to LBSM data to gain a more fine-grained understanding of the urban environment [63, 71]. This approach allows researchers to move beyond conventional area-based geographic boundaries, such as census tracts and buffer zones, which provide a limited view of individuals' food choices and misrepresent the reality of their perceived food environment and dietary patterns [72]. Instead, researchers are using LBSM data to analyze food environments based on location points tagged through social media posts [62, 73]. By using LBSM data to analyze the urban environment and understand the interactions between citizens and urban spaces, health geography researchers have new opportunities to gain insights into public health.

However, previous food environment studies focused primarily on the spatial distribution of healthy and unhealthy foods relying on common sense categorizations. For instance, words like "vegetables" were considered healthy, while words like "french fries" were considered unhealthy [62, 63]. To expand the analysis of the urban food environment using LBSM data, researchers need a more comprehensive list of food-related words with their associated healthiness degrees.

Although some studies have examined opinion word expansions [74], few have focused specifically on food word expansion for geographic analysis with LBSM data. To address this research gap and establish a foundation for urban food environment studies using LBSM data in health geography, this research proposes a novel method for expanding food-related words and predicting their food energy density based on machine learning and word embedding techniques.

## Methods

### Energy density classification

We classified food words based on its energy density (ED), which refers to the amount of energy or calories in a given weight of food, typically measured in kilocalories per gram. Foods with lower ED contain fewer calories per gram than those with higher ED [75]. The British Nutrition Foundation classification system divides foods into four levels based on their ED: very low, low, medium, and high. Foods with an ED lower than 0.6 kcal/g are considered very low, while those with an ED between 0.6 and 1.5 kcal/g are considered low. Foods with an ED between 1.5 and 4 kcal/g are classified as medium, and those with an ED above 4 kcal/g are classified as high. According to the British Nutrition Foundation and previous studies, a healthy diet should consist mainly of very low and low ED foods, while moderate consumption of medium ED foods and limited consumption of high ED foods [76].

In this study, we classified foods into two categories based on their ED. Foods with very low and low ED were classified as "L-ED", while those with medium and high ED were classified as "H-ED". Table 1 shows the new classification system. It is important to note that in this study, "L-ED" and "H-ED" refer specifically to their association with the prevalence of obesity, and not general health. To avoid confusion, we will use "L-ED" and "H-ED" to describe the two classifications of the foods thereafter.

**Table 1:** New classification based on the British Nutrition Foundation classification

British nutrition foundation			New classification	
Classification	Energy density (ED in kcal/g)	Example words	Classification	Energy density (ED in kcal/g)
Very Low	ED $<$ 0.6	Asparagus; cabbage	L-ED	ED $<$ 1.5
Low	0.6 $\le$ ED $<$ 1.5	Edamame; haddock		
Medium	1.5 $\le$ ED $\le$ 4	Breadstick; chicken	H-ED	ED $\ge$ 1.5
High	ED $>$ 4	Potato chips; peanut butter		

### Data and preprocessing

To build the food word dictionary, we started by compiling an initial set of food words, or "seed words", from the United States Department of Agriculture (USDA) food composition database^1^ and the Open Food Facts (OFF) database.^2^ The USDA database contains detailed nutritional information on 7524 food items, while the OFF is a crowdsourced website with nearly 665,000 user-reported food items with nutritional information, including food words from different cultures, such as French food items and other international food brands not commonly found in official food reports. This is particularly important for a multicultural city like Toronto, which has a diverse range of food outlets.

To ensure accuracy, data was cleaned to remove duplicates, and food items were manually checked to eliminate any non-identifiable foods and brands. USDA food words were preliminarily cleaned and combined to ensure their suitability for natural language processing. The USDA dataset contains many detailed keywords, so similar food words were combined into one representative word. For example, all types and brands of beer in the database were aggregated into one item listed as 'Beer', and the mean energy density was calculated. Following preprocessing, 967 food words were extracted from the USDA database and categorized as L-ED or H-ED based on their mean energy density.

On the OFF website, all food products are described by their label name on sale and their nutrition facts label. With millions of listed food products, the Natural Language Toolkit^3^ was used to clean the data. All punctuation and non-word characters were removed, and food items lacking energy density information were excluded from further processing. After the initial cleaning, food words were selected from the OFF database to complete the initial seed words dictionary.

**Fig. 1:**
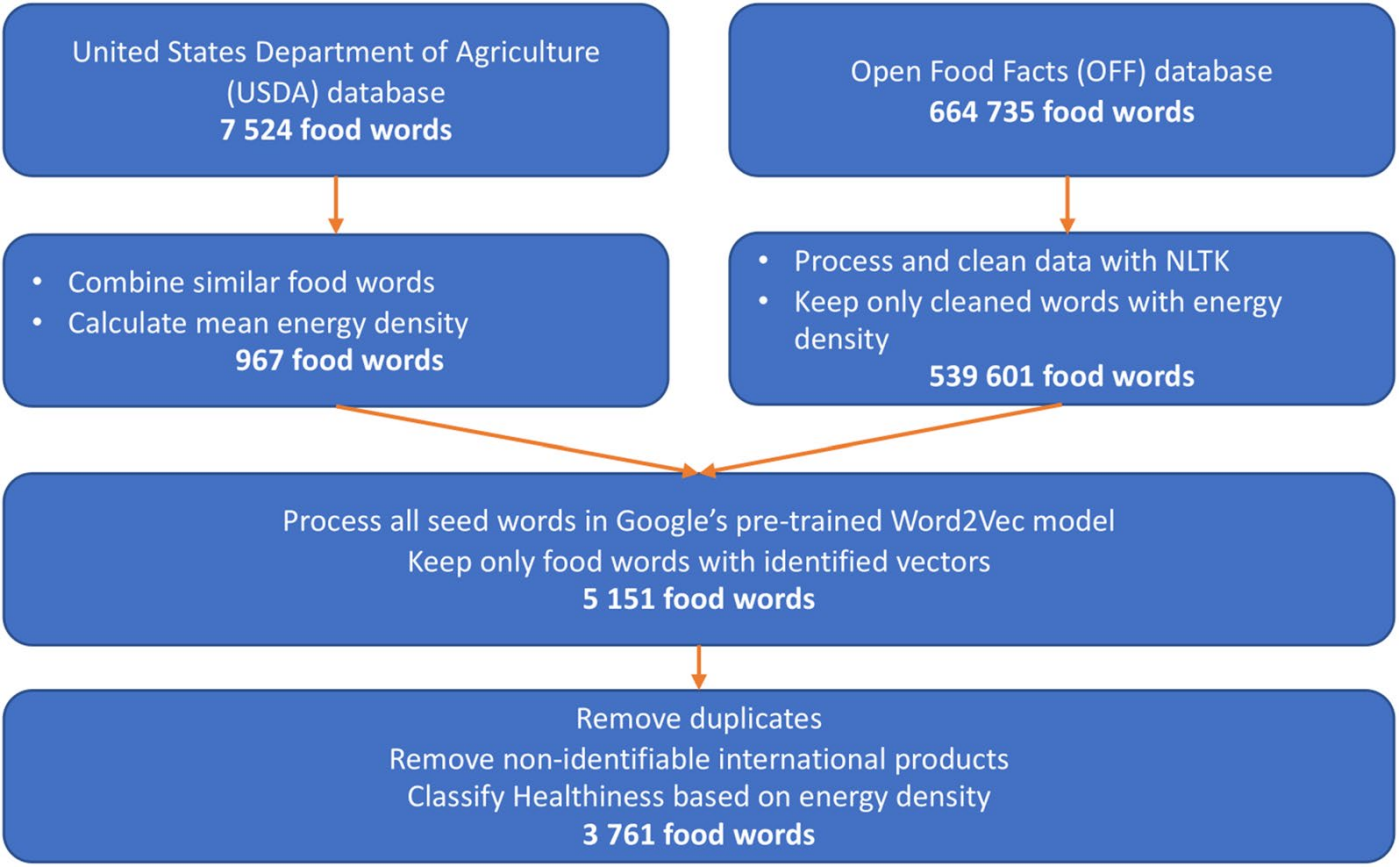
The workflow of the data preprocessing

To expand the food words from these existing food words, we utilized the embedded vectors of each word to find new, similar food words in the embedding space. Embedded vectors (also known as word embedding), are utilized in natural language processing to depict words as numeric value vectors, capturing the sematic relationship between them. [77] These vectors, typically ranging from 100 to 300 dimensions, are generated by unsupervised learning algorithms (e.g., Word2Vec) that analyze the co-occurrence patterns of words from large text data sets. [78] The embedding space is the multi-dimensional vector space where words are mapped as embedded vectors. Each dimension in this space corresponds to a specific feature or aspect of the word's meaning. In the embedding space, words with similar features tend to have vectors that are close to each other, while dissimilar ones are farther apart. Google's pre-trained Word2Vec^4^ model was employed as the word embedding space for the preprocessed seed words. This model includes a vocabulary of almost 3 million words and phrases, trained on roughly 100 billion words from the Google News dataset. Each word is represented by 300 feature vectors in the embedding space. Out of the combined preprocessed seed words from the USDA and OFF databases, 5151 words can be found in the embedding space. Finally, after removing duplicates and non-retrievable food products, we obtained 3761 food words associated with energy density values are set as the initial dictionary of food words. Figure 1 illustrates the workflow of the data preprocessing.

### Machine learning models for food energy density prediction

In daily language usage, high ED food words (e.g., burgers, fries, etc.) tend to appear together, while low ED food words (e.g., fruits, vegetables, etc.) cluster closely. While there may be some exceptions and complexities, food words with similar attributes (e.g., energy density) may be clustered with multiple centroids in the hyper-dimension word embedding space. Therefore, we used 300 feature vectors of the cleaned 3761 seed food words as prediction variables and their food energy density classification (i.e., L-ED or H-ED) as the target variable to train machine learning models.

To ensure a comprehensive exploration of the prediction problem, we tested ten widely used machine learning models. The models include Artificial Neural Network (ANN), Support Vector Machine (SVM), Gaussian Process (GP), AdaBoost (AB), Naïve Bayes (NB), Quadratic Discriminant Analysis (QDA), Gradient Boosting (GB), k-Nearest Neighbors (KNN), Random Forest (RF), and Decision Tree (DT). The selected models are widely recognized in machine learning literatures and have demonstrated successful application in a variety of domains. Each model represents a distinct approach and has its own unique strengths and assumptions about the data. Furthermore, these models include a wide range of machine learning families that are designed with different paradigms, which enables a thorough comparison of different algorithmic approaches. By evaluating multiple models, we increase the chances of finding the one that performs exceptionally well for the food words prediction task. To determine the model that predicts the food energy density category with the highest accuracy, we tested these models using fivefold cross-validation.

### Food word expansion based on cluster analysis of word embedding space

In order to expand the food word dictionary by discovering new words, we follow the assumption that food words with similar characteristics will exhibit certain patterns in the word embedding space [79]. This means that similar words can be clustered together and form clusters in the word embedding space based on the different food groups they belong to. We also expect these clusters to follow a finite number of Gaussian distributions in the word embedding space. To analyze and discover these clusters, we used a probabilistic model known as the Gaussian mixture model. This model attempts to represent normally distributed subpopulations within a normally distributed overall population [80]. It assumes that the data points follow patterns of several clusters with a Gaussian distribution, making it an ideal method to analyze and discover new food words around the centroids of these clusters.

Using the Gaussian mixture model on the initial food seed words, we created a probabilistic field with several cluster centers in the word embedding space. The probability level determines the likelihood that a word in the embedding space belongs to a discovered cluster. At a given probability level, multiple hyperellipses are formed in the word embedding space, where all the words contained inside these hyperellipses can be similar food-related words within the range of that specific probability level. Therefore, the higher the probability level, the higher the chance that the newly discovered words are food-related, but a smaller number of new words can be discovered.

In order to reduce the computation time required to search for new words in the embedding space, we also set a similarity level in conjunction with the probability level. The similarity level is the cosine distance between two locations in the word embedding space. By setting this similarity level, we can limit the number of words tested to see if they belong within the same probability level. We use the Genism^5^ Python library to extract all the words within a specific similarity level from the discovered cluster centroids. We then test these words one by one to determine whether they are distributed inside these hyperellipses with a specific probability level. We try different probability and similarity level combinations and compare the results. Finally, the accuracy of the expanded food words being related to food words is verified by human interpretation with a random subset of the expanded food words.

There is a trade-off relationship between accuracy and the number of newly discovered food words. When adjusting the similarity and probability levels, we found that there is a balance between accuracy and the number of new words identified. If we set similarity and probability levels low, the accuracy of the results decreases by incorrectly identifying food words. Conversely, setting the levels high results in a reduced number of newly discovered words, as the model becomes overly conservative. Therefore, finding an optimal balance is crucial to strike a trade-off between achieving high accuracy and maximizing the number of food words discovered.

## Results

### Food energy density prediction model

The first step was to test various machine learning models to determine the most accurate predictor of the food energy density level of the food words. The 300 feature vectors of the seed words were used as the prediction variable, and their food energy density level was used as the target to train the different machine learning models. The Scikit-learn^6^ module for Python was employed to train the model with default parameter settings for the initial selection. Table 2 lists the mean accuracy and associated standard deviation of the fivefold cross-validation of the ten different machine learning models used to predict the food energy density of food words.

**Table 2:** The mean accuracy and standard deviation of fivefold cross-validation of the machine learning models

Machine learning models	Mean accuracy (Std. Dev.) (%)
Artificial Neural Network (ANN)	87.55 (7)
Support Vector Machine (SVM)	90.74 (7)
Gaussian Process (GP)	85.70 (4)
AdaBoost (AB)	59.24 (30)
Naïve Bayes (NB)	67.44 (18)
Quadratic Discriminant Analysis (QDA)	78.12 (5)
Gradient Boosting (GB)	62.82 (23)
k-Nearest-Neighbor (KNN)	84.86 (3)
Random Forest (RF)	82.51 (9)
Decision Tree (DT)	30.43 (23)

Of the ten models, ANN, SVM, GP, KNN, and RF, exhibited the highest prediction accuracies, with the SVM (90.74% accuracy) ranking number one. These five models were further evaluated with hyperparameter tuning to optimize their hyperparameters (results are in Table 3). Machine learning models normally have different setting parameters known as hyperparameters that control their learning process. Prior to training a model, it is crucial to define these hyperparameters. By systematically exploring different combinations of hyperparameter values, the optimal configuration can be determined when the model achieves the highest accuracy. This procedure is commonly referred to as hyperparameter tuning. From the tuned models, the SVM still achieved the highest mean accuracy (an increase to 91.62% from 90.74%) in correctly predicting the food energy density classification of the food words.

**Table 3:** The mean accuracy and standard deviation of fivefold cross-validation of the machine learning models before and after hyperparameter tunning

Machine learning models	Mean accuracy before tuning (%)	Mean accuracy after tuning (%)
ANN	87.55	89.10
SVM	90.74	91.62
GP	85.69	85.86
KNN	84.86	85.29
RF	82.51	83.69

### Food word clustering in word embedding space

The clustering analysis of the initial seed words was carried out using the Gaussian mixture model. The model has four options to constrain the covariance between the estimated classes, which determines the degree of freedom in shape, length of axes, and direction of all the ellipsoids of formed clusters. This hyperparameter includes "diagonal," "tied," "full," and "spherical." Additionally, the number of components (clusters) needs to be defined, with which the algorithm will form the clusters in the given number. The number of clusters and covariance type need to be calibrated to find the best-fit model. We iterated the four covariance types and the number of clusters to train the model and compared their results with the AIC values. The best model performance (with the lowest AIC value) is achieved when the number of clusters equals 36 (see Additional file 1 for more details of the calibration).

### Food words expansion and food energy density prediction

**Fig. 2:**
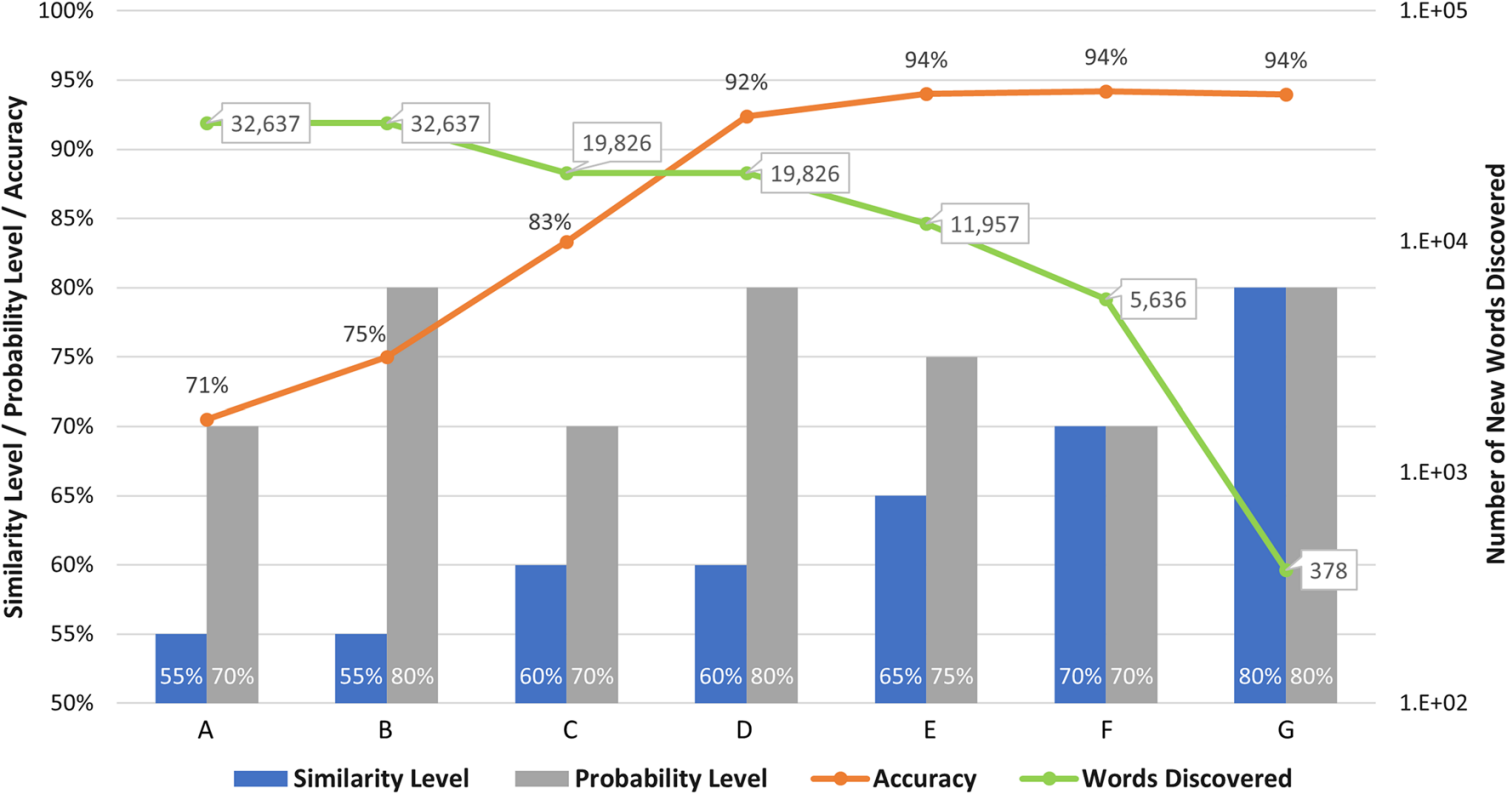
Number of new words discovered with different similarity and probability levels, and their respective accuracy rates achieved

The results of the Gaussian mixture model indicated that 36 clusters were identified from the seed words. Using the centroids of the clusters, we tested different combinations of probability and similarity levels to determine which one produced the best likelihood of the newly discovered words being food-related. For each set of food word expansion tested, we randomly selected 100 words and evaluated their accuracy in identifying food-related words through human interpretation. We also assessed the percentage of existing L-ED and H-ED food words among the newly discovered food words. Table 4 and Fig. 2 present the results of the word expansion using distinct similarity and probability configurations. Within both the table and figure, the alphabetic labels A to G represent diverse expansion scenarios, each characterized by a unique combination of similarity and probability settings.

**Table 4:** Diverse Expansion Scenarios: Results of Word Expansion Using Varied Similarity and Probability Settings

	Food word expansion scenarios						
	A	B	C	D	E	F	G
Similarity Level	0.55	0.55	0.6	0.6	0.65	0.7	0.8
Probability Level	0.7	0.8	0.7	0.8	0.75	0.7	0.8
Number of expanded words	32,637	32,637	19,826	19,826	11,957	5,636	378
Accuracy	71%	75%	83%	92%	94%	94%	94%
Percentage of L-ED food words	6.08%	6.08%	5.98%	5.98%	5.02%	4.67%	5.82%
Percentage of H-ED food words	93.92%	93.92%	94.02%	94.02%	94.98%	95.33%	94.18%

The columns labeled with alphabetic identifiers A to G depict distinct expansion scenarios, each defined by a unique combination of similarity and probability levels

The results show that as the similarity and probability levels increase, fewer words are discovered, but with a higher likelihood of being food words. Conversely, lower similarity and probability levels result in more words discovered, but with a lower chance of them being food words. The highest number of food words discovered was 32,637 words, which was obtained with a combination of a 0.55 similarity level and a 0.7 probability level (food word expansion A). This combination resulted in an accuracy of 71% of the 32,637 words being food-related words. However, a slightly higher accuracy of 75% was achieved with a higher probability level of 0.8 (food word expansion B) for the same 0.55 similarity level. Although these combinations yielded the highest number of newly discovered words, they obtained the lowest accuracy scores out of all similarity and probability level combinations.

In terms of food-word accuracy, the highest accuracy score of 94% was obtained in three situations. First, with the highest level of 0.8 similarity and probability levels (food word expansion G), only 378 new words were discovered, marking it as the combination with the lowest number of words discovered. At the 0.7 similarity and probability level (food word expansion F), the number of new words discovered increases to 5,636 while maintaining an accuracy of 94% that the new words are food-related words. Lastly, for the same accuracy score, the combination of a 0.65 similarity level and 0.75 probability level (food word expansion E) achieved the highest number of new words discovered, with 11,957 new words. Other combinations (food word expansion C and D) yielded a higher number of new words discovered, with 19,826 new words, but scored lower accuracy of 83% (food word expansion C) and 92% (food word expansion D). Considering all different combinations, we determined that food word expansion E achieved the best results with a relatively high number of new words discovered (11,957 new words) while maintaining a high accuracy score of 94% for the new word being food-related.

With the newly discovered food-related words and their 300 feature vectors, we used the trained SVM model to predict the food energy density classification of these new words. Given that the optimized SVM model achieved a mean accuracy of 92% with fivefold cross-validation in predicting the food energy density classification of the initial seed words, we can assume that the new classification predictions of the newly discovered food words will also yield a similarly high level of accuracy.

Table 5 presents examples of the food energy density classification predictions of new food words obtained with the SVM model. The predictions seem to correctly classify the new words, where more fruit- and vegetable-related words (i.e., lemon peel, basil, persimmons) are classified as L-ED, and meat and processed foods are categorized as H-ED.

**Table 5:** Examples of newly discovered food words and their predicted food energy density classification (1: L-ED; 2: H-ED) at a 0.65 similarity level and a 0.75 probability level

Newly discovered words	Food energy density classification prediction
Banana_cream_pie	2
Blueberry_pancakes	2
Lemon_peel	1
Basil	1
Bbeef_stew	2
Coconut_milk	1
Kfc	2
Samosas	2
Tomato_salad	1
Sweet_potato_fries	2
Braised_lamb	2
Blueberry_muffins	2
Jambon	2
Raclette	2
Balsamic_vinegar	1
Mango_peach	1
Fried_calamari	2
Beef_shawarma	2
Gnocchi	2
Persimmons	1
…	…

With the addition of the newly discovered food words, the expanded food word dictionary consisted of 14,152 food words that were classified by food energy density. Additionally, the food word dictionary contained food words with varying numbers of words. For instance, 2-word food words (e.g., chocolate chip), 3-word food words (e.g., sweet potato fries), and 4-word food words (e.g., freshly squeezed lime juice), were present in the dictionary. Table 6 demonstrates that the expanded food word dictionary exhibited a significant increase, particularly in the multi-word food words. The variety of food words enables more precise descriptions of food, and more crucially, enables the identification of words that describe diverse dishes, highlightingredients included.

**Table 6:** Comparison of the food words between the original and expanded dictionary

	1-word food word	2-word food word	3-word food word	4-word food words	Total
Original food words	1609	1835	303	14	3761
Newly discovered food words	1694	7926	2186	151	11,957
Example words	Macaron; Tapenade	Chocolate fudge; Lemon peel	Cinnamon toast crunch; Vanilla ice cream	Freshly squeezed orange juice	
Percentage of increase	2.57%	62.40%	75.65%	83.03%	52.14%

## Case study: yelp reviews analysis

To evaluate the performance of the expanded dictionary, which contains new food words with their food energy density classification, in the context of food environment studies, we compared and tested the datasets using Yelp reviews to analyze the food words mentioned by reviewers on food outlets in the City of Toronto. Toronto is the largest municipal jurisdiction in Canada and the fourth most populated city in North America. It is renowned for being one of the most multicultural cities in North America, with almost half of its residents being immigrants born outside of the country, contributing to a diverse food environment.

The data were collected from March 2019 to March 2020 for all food outlets listed on Yelp in the City of Toronto. We identified a total of 5,855 of the most reviewed food outlets from Yelp, excluding grocery stores, to investigate the city's food environment. We analyzed the six districts of the city: East York, Etobicoke, North York, Old Toronto, Scarborough, and York. We compared differences in the quantity of identified words and further performed Pearson's t-tests to evaluate if the differences in words identified with the expanded dictionary showed a significant improvement over the original dictionary.

**Fig. 3:**
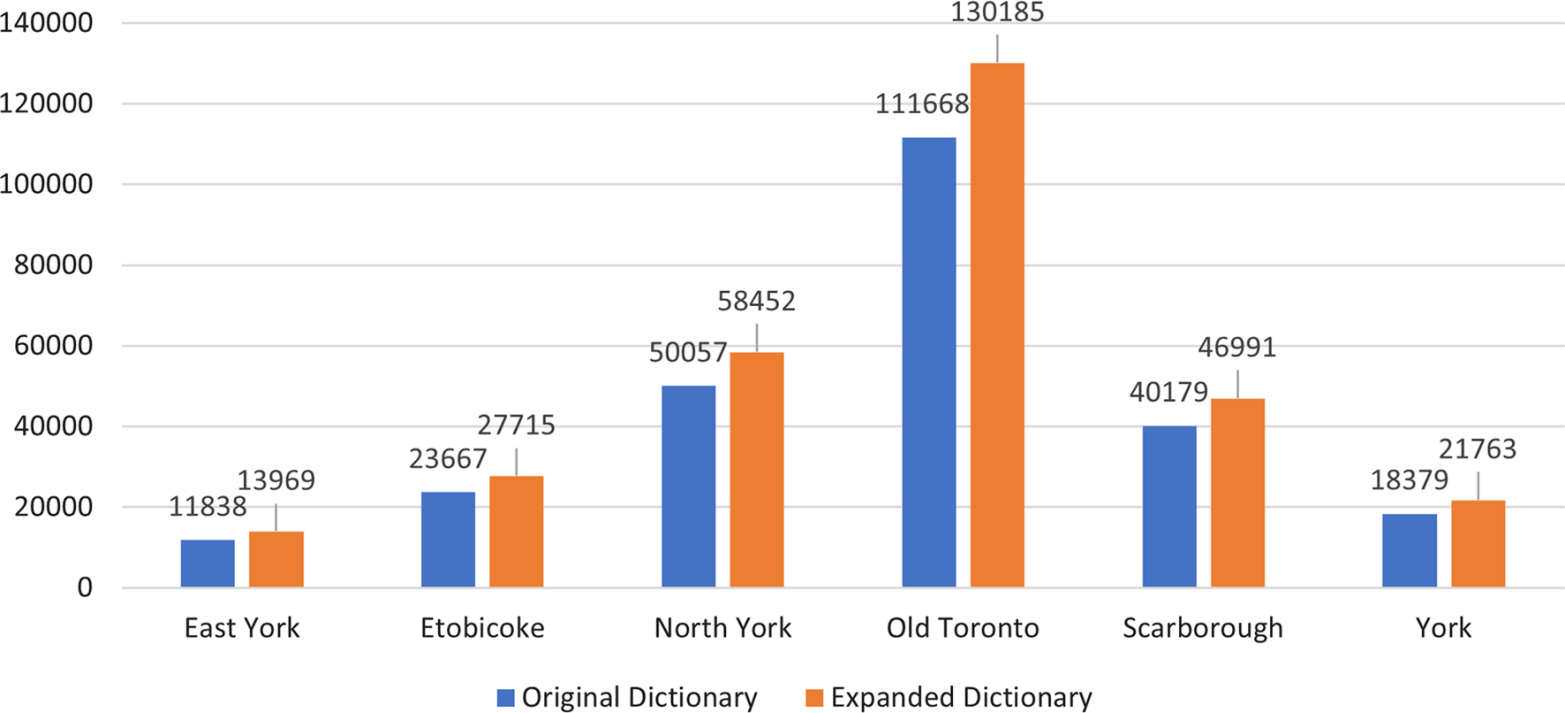
Number of food words identified in Yelp reviews with the original and expanded dictionaries in the six districts of Toronto

The results from Table 7 and Fig. 3 demonstrate that the expanded food word dictionary was able to identify more food words in the Yelp reviews for all districts in Toronto. Specifically, the expanded dictionary yielded an increase of more than 7% in the number of food words identified. Pearson's t-tests were then conducted to assess the significance of the difference in the number of words identified with the expanded dictionary compared to the original dictionary, as shown in Table 8. The results indicate that the difference is significant for the North York and Old Toronto districts, which have the highest number of food outlets. However, the significance of the difference may vary due to the variation in the number of food outlets and reviews across districts.

**Table 7:** Total number of food words identified in Yelp reviews of food outlets in the districts of Toronto

Districts	East York	Etobicoke	North York	Old Toronto	Scarborough	York
Number of Food Outlets	487	746	1,194	2219	797	412
Number of Words Identified with the Original Dictionary	11,838	23,667	50,057	111,668	40,179	18,379
Number of Words Identified with the Expanded Dictionary	13,969	27,715	58,452	130,185	46,991	21,763
Percentage of Increase in Words Identified	8.26%	7.88%	7.74%	7.66%	7.81%	8.43%

**Table 8:** T-test comparing the analysis results of Yelp reviews based on the expanded and original food word dictionary

Districts	df	t-value	p-value
East York	946.15	− 1.578	0.115
Etobicoke	1452.1	− 1.697	0.090
North York	2327.7	− 2.126	0.034*
Old Toronto	4329.1	3.276	0.001**
Scarborough	1554.1	− 1.839	0.066
York	796.62	1.364	0.173

* p < 0.05, ** p < 0.01

Since Yelp reviews reflect consumers' experiences with food outlets, the abore descriptive food words associated with specific cuisines and different types of meals is essential for analyzing the culturally diverse urban food environment in Toronto. As presented in Table 9, the expanded dictionary was able to detect more multi-word food words, with 2-word food words showing an increase of 34.69% and 3-word and 4-word food words showing increases of over 50%.

**Table 9:** Number of food words identified in the Yelp reviews with the original and expanded dictionary

	1-word food words	2-word food words	3-word food words	4-word food words
Original Words	87,669	10,733	432	5
Expanded Words	91,383	22,137	1411	16
Percentage increase in words identified	2.07%	34.69%	53.12%	52.38%

Further analysis was conducted using Pearson's t-tests to identify significant differences in the number of words discovered between the original and expanded food word dictionaries for two-word and three-word food words. The results in Table 10 show that the expanded dictionary captured significantly more food words than the original for both two-word and three-word combinations.

**Table 10:** T-test comparing the analysis results of Yelp reviews based on the expanded and original food word dictionary

	df	t-value	*p*-value
1-word Food Words	67.887	0.140	0.889
2-word Food Words	48.681	2.294	0.026*
3-word Food Words	41.114	3.247	0.002**
4-word Food Words	49.175	1.888	0.065

* p < 0.05, ** p < 0.01

**Fig. 4:**
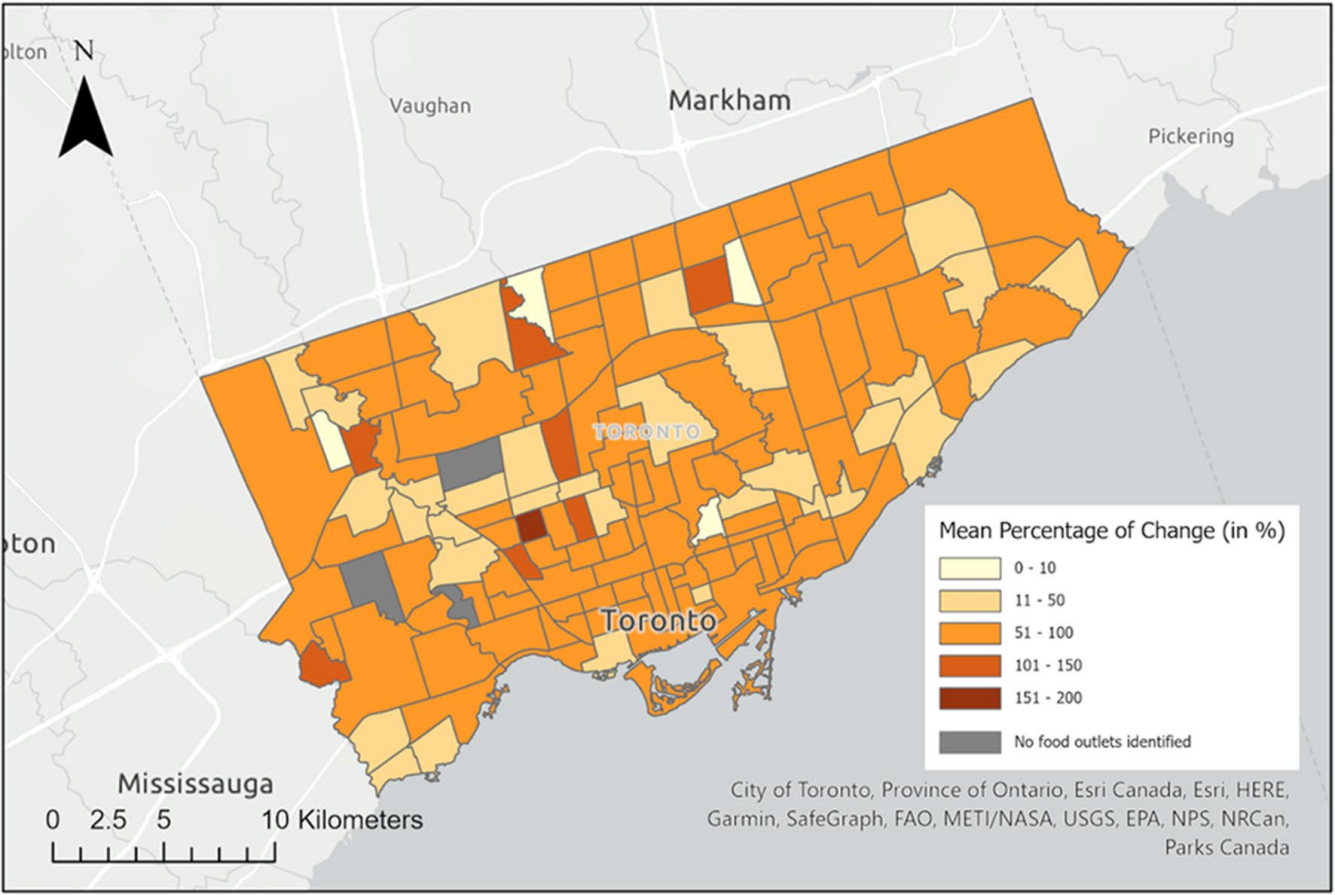
The difference in densities of food words identified per food outlet with the original and expanded dictionary by the mean percentage of change in the number of 2-word food words discovered by neighbourhood in Toronto

Figure 4 displays the mean percentage of change in the number of 2-word food words identified before and after the expanded dictionary implementation. The map illustrates a significant change, with most neighbourhoods exhibiting a more than 50% increase. However, some residential-only neighbourhoods, such as Princess-Rosethorn, Maple Leaf, and Lambton-Baby Point, have few food outlets and thus no food words were identified in these areas during the study.

**Fig. 5:**
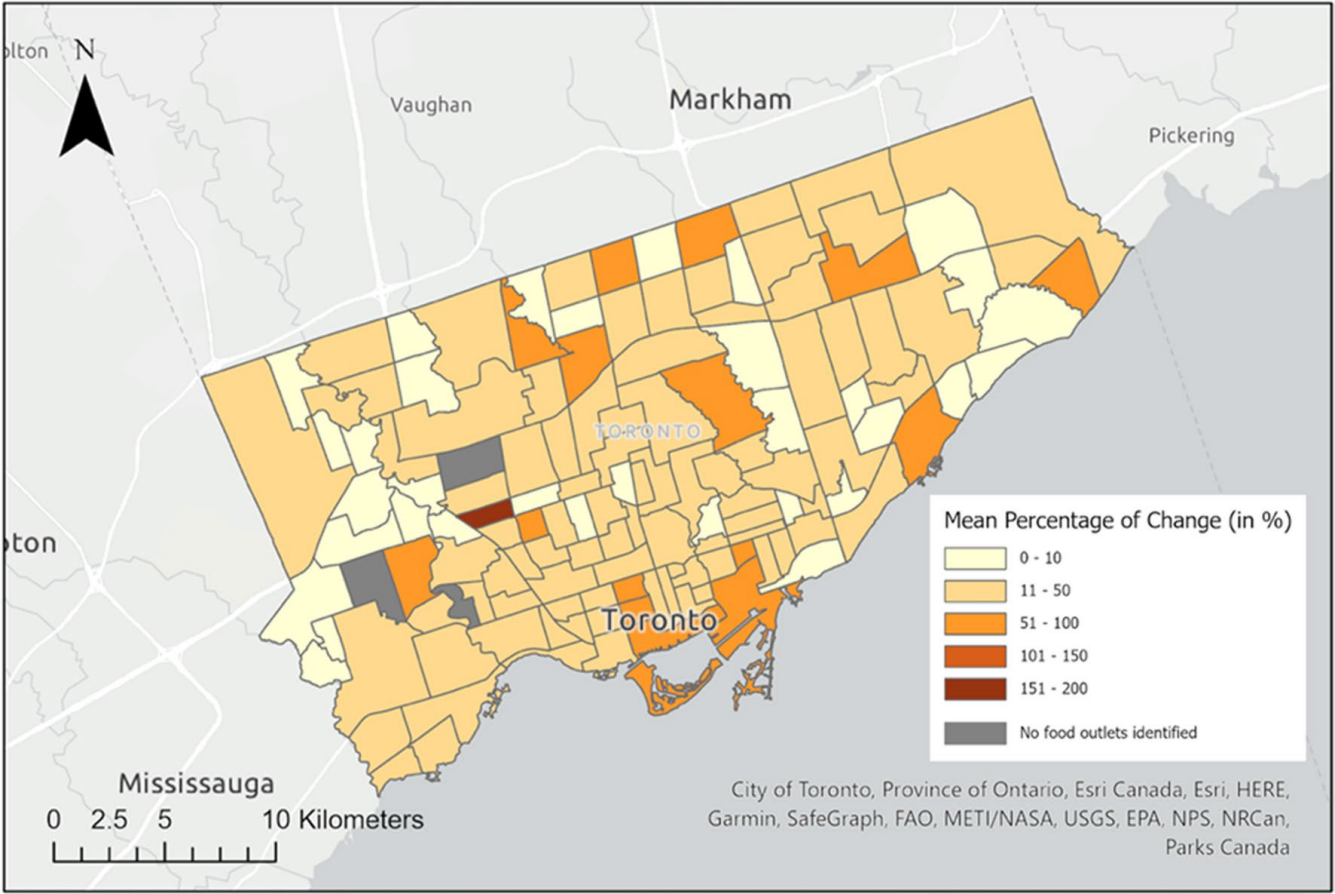
The difference in densities of food words identified per food outlet with the original and expanded dictionary by the mean percentage of change in the number of 3-word food words discovered by neighbourhood in Toronto

In terms of density, although the number of 3-word food words identified per food outlet was much lower than that of 2-word food words, Fig. 5 shows that the expanded dictionary still yielded a higher number of food words per food outlet. Most neighbourhoods can be seen to have an 11% to 50% increase in the number of 3-word food words identified, and some neighbourhoods showed a significant increase between 51 to 100%. Despite the lower density, the expanded dictionary significantly improved the detection of more 3-word food words in the reviews compared to the original dictionary.

**Fig. 6:**
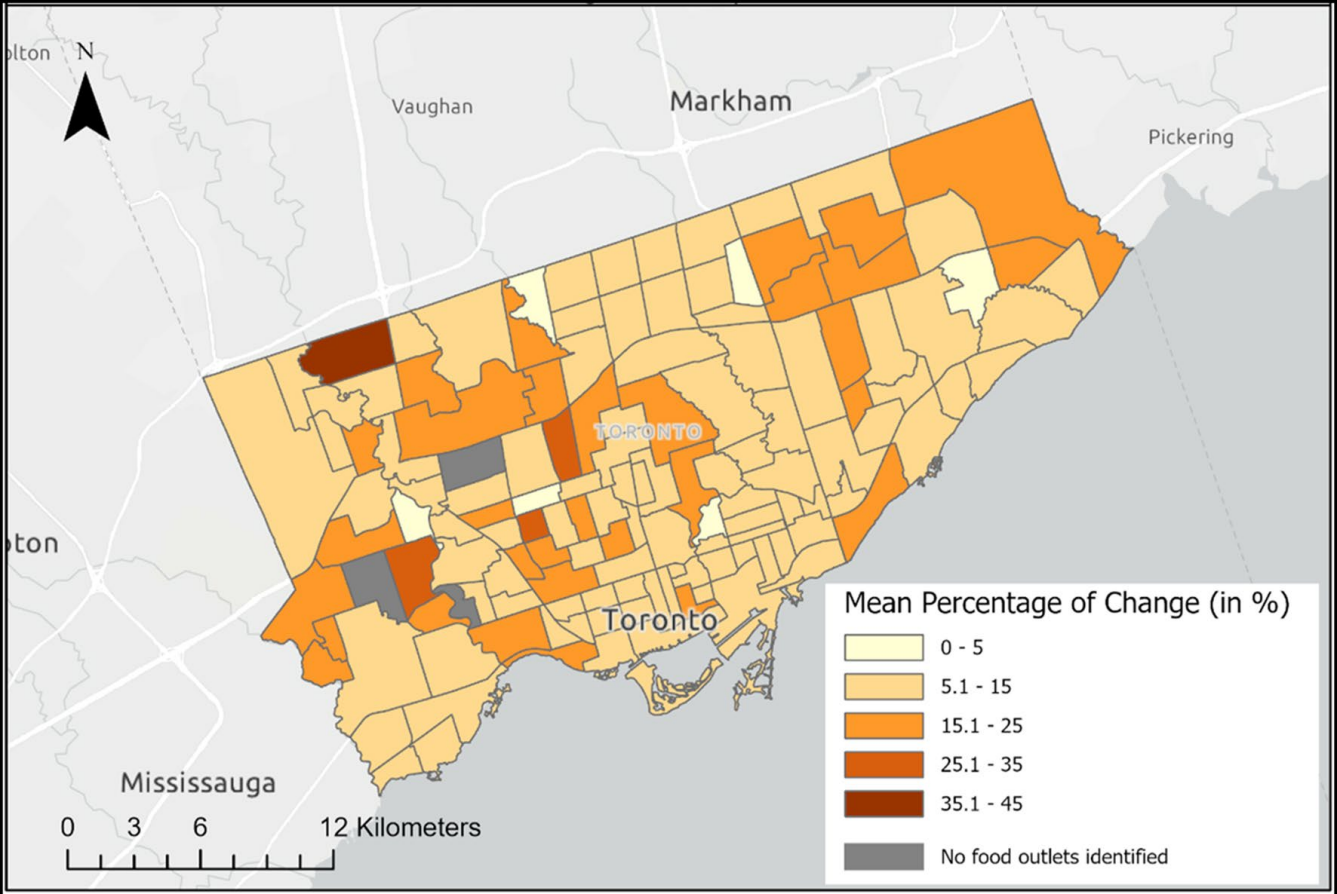
The difference in densities of food words identified per food outlet with the original and expanded dictionary by the mean percentage of change in the total number of food words discovered by neighbourhood

Finally, Fig. 6 displays the differences in the density of food words identified by neighbourhood between the original and expanded dictionaries. The majority of neighbourhoods showed an increase of between 5 to 15% in the number of food words detected, with some neighbourhoods recording an increase of over 15%.

The mean percentage change depicted in Figs. 4, 5, 6 highlights the disparity in the densities of identified food words per food outlet between the original and expanded food word databases. This comparative analysis exposes the potential inaccuracies that may arise when relying solely on the unexpanded food words database, as a significant number of food words could be overlooked. By examining the spatial distribution of food words with different energy density, we could gain valuable insights into the prevalence and prominence of various food categories within a specific area or time period. Any observed shifts over time may indicate changes in food consumption patterns, the impact of environment or policy factors on food choices, and potential disparities in food access. Such analyses can contribute to a better understanding of evolving dietary patterns, enabling informed decisions to enhance public health.

## Discussion

In this study, we collected initial seed words from both an official list of food items extracted from USDA and a crowdsourced database containing multicultural food products extracted from OFF. This allowed us to expand the food word dictionary, which had previously been limited to stereotypical food words representing the healthiness of foods (e.g., fruits and vegetables, fast food items). Building the initial food word dictionary based on these sources was beneficial because it included multicultural food items not commonly included in North American food environment studies.

The compilation of food words from the OFF database brought an assorted of food words not typically found in official reports. Because the information on food items is crowdsourced, we can assume that the users contributed food items are what they typically consume. Furthermore, using Google's Word2Vec model, which is trained on Google's News platform, we were able to include more descriptive variations of food items, resulting in an expansive and descriptive range of food words in the expanded food word dictionary.

Expanding the food word dictionary is a balance of accuracy and quantity. The Gaussian mixture model allowed us to find clusters of food-related words in the hyper-dimension word embedding space. We discovered new words from those clusters using two parameter settings: similarity and probability. The higher the similarity and probability settings, the fewer words were discovered, but with a relatively higher chance of the words being food words (higher accuracy). Conversely, the lower the similarity and probability levels, the higher the number of words discovered, but the chance that these words were food words was relatively lower. In this preliminary study, we chose the settings of a 0.65 similarity level and a 0.75 probability level, resulting in the discovery of 11,957 new words while still maintaining an accuracy of 94%. However, the minimum accuracy levels or the target number of words to be discovered may differ in specific applications, which must be flexibly adjusted accordingly.

Machine learning models were established to predict the food energy density of newly discovered food words. The prediction models were trained by the initial seed words dictionary associated with ED values. Seed words were classified by the food energy density according to the ED of the food, considering that food environment studies are mainly focused on health issues related to the prevalence of obesity. Based on the British Nutrition Foundation's categorization, four levels of classification (very low, low, medium, and high) were categorized into two categories by grouping very low and low into one classification (L-ED), and medium and high into another (H-ED). This division followed dietary recommendations that encouraged the consumption of relatively low ED foods while consuming relatively high ED foods in moderation. In our model, the 300 feature vectors (in the word embedding space) of the seed words were set as the prediction variables, while the food energy density classification (L-ED or H-ED) acted as the target variable to train the machine learning models. Among the different models being tested, the SVM model yielded the highest accuracy in predicting the classification. This model was able to predict the classifications of the new words with a relatively high accuracy of 91.62%.

We tested the newly compiled food dictionary, which contains a total of 14,152 food words and their predicted food energy density categories, on Yelp reviews of food outlets in the City of Toronto to compare the number of food words identified. Results showed that the expanded food dictionary identified many more food words compared to the original dictionary. Further analyses showed that the expanded dictionary was especially effective in identifying 2-word food words and 3-word food words. This finding is particularly valuable in understanding the food environment, as the expanded food word dictionary can capture a variety of descriptive dishes found on restaurant menus and different types of cuisines that would otherwise not be identifiable with simple food words.

The positive results of these preliminary analyses on food outlets within the city's districts and neighbourhoods suggest that this food word expansion could further assist spatial analyses in food environment studies. Further studies utilizing social media data to investigate the spatial component of food environments could benefit from the addition of this food word expansion. Additionally, the expanded dictionary can be used for sentiment analysis on Yelp reviews to evaluate the emotional tone of the reviews towards different food outlets. This can provide insights into how the food environment and its related words affect people's emotions towards the food outlets in different districts of Toronto. This can be useful for understanding the food environment's impact on people's overall well-being and the potential for improving it through promoting healthier food choices. Overall, the expanded food word dictionary can provide valuable information for food environment studies and interventions.

The proposed method for food word expansion and food energy density prediction can be used to analyze the urban food environment using LBSM data, providing insights into the urban environment and the interactions between citizens and urban spaces. This technique could also be used in other studies using LBSM to understand the urban environment, such as the friendliness of physical activity and the utility of urban green space. The results of the urban environment analysis based on the proposed word expansion method can help urban planners and city managers better understand the city and serve their citizens.

Beyond analyzing the food and urban environment, our modeling approach has significant implications for public health policy. By identifying the prevalence of low and high energy density foods, we can gain insights into the overall food environment and its evolving over time. The findings can inform public health strategies, such as targeted interventions to promote the availability and accessibility of low energy density foods or initiatives to educate and empower individuals to make healthier food choices. Additionally, by comparing the food environment before and after an implementation of a health policy, the model can help policymakers in evaluating the effectiveness of the policy to create supportive environments that foster healthier eating habits and combat diet-related diseases.

Although the expanded food word dictionary demonstrated promising results in identifying food words, there are several limitations to consider. First, the initial seed words used may be limited to the context of North America, and food words from other cultures or languages that are not popular in North America may not be included in the expansion. Thus, applying the expanded food words dictionary may require further validation if used in regions other than North America. Second, the Word2Vec model used in this study is trained based on Google News, which may not capture informal expressions of foods used in daily life. Finally, although the expanded food words dictionary was tested on the Yelp reviews analysis with good results, further validation is still needed with other social media data for food environment analysis.

## Conclusion

This study proposes a novel method to expand food-related vocabulary and predict the food energy density based on machine learning and word embedding. This method makes a valuable contribution to building a more comprehensive list of food words that can be used in geography and public health studies by mining geotagged social media data. Previous studies categorized food items based on the common understanding of their healthiness, but this study used the ED to categorize foods, which allowed for a wider variety of food items to be included. The final food word dictionary included an array of descriptive dishes, specific ingredients, and cooking methods, including international food products and brands. This advancement is significant because it enables the portrayal of a diverse and multicultural food environment that is not limited to stereotypical healthy and unhealthy foods, thereby providing a better understanding of the spatial disparity of food environment and its evolving over time.

The results of this study provide a foundation for future urban food environment studies using widely available social media data. By employing the proposed modeling approach, we can expand our understanding of the food environment, identify emerging trends, and pinpoint areas where interventions can have the most impact. Ultimately, the aim is to improve population health outcomes by promoting healthier diets and reducing the burden of diet-related diseases.

### Supplementary Information

**Additional file 1:** **Fig. S1. **The initial calibration of the Gaussian mixture models (the vertical axis represents the AIC value of the model; the horizontal axis represents the number of clusters from 1 to 101 with a step of 5). **Fig. S2. **The further calibration of the Gaussian mixture models (the vertical axis represents the AIC value of the model; the horizontal axis represents the number of clusters from 20 to 60 with a step of 1). **Fig. S3** Location and Spatial Boundaries of Toronto's Six Districts.

## Data Availability

The datasets supporting the conclusions of this article are available in the U.S. Department of Agriculture, the Open Food Facts, and Yelp.com.

## Footnotes

1 Standard Reference of food composition originally available via the USDA National Nutrient Database—final release April 2018 (https://fdc.nal.usda.gov/download-datasets.html).

2 Open Food Fact database (https://world.openfoodfacts.org/data).

3 Natural Language Toolkit is a “platform for building Python programs to work with human language data” (https://www.nltk.org/index.html).

4 Word2Vec “provides an efficient implementation of the continuous bag-of-words and skip-gram architectures for computing vector representations of words” (https://code.google.com/archive/p/word2vec/).

5 https://github.com/RaRe-Technologies/gensim.

6 https://scikit-learn.org.
