# Supplementary material for: Empowering health geography research with location-based social media data: innovative food word expansion and energy density prediction via word embedding and machine learning
Source: Int J Health Geogr. 2023 Sep 16;22:22. doi: 10.1186/s12942-023-00344-5 (PMC10505329; doi:10.1186/s12942-023-00344-5)
Supplement: Supplementary file 1 — Additional file 1: Fig. S1. The initial calibration of the Gaussian mixture models (the vertical axis represents the AIC value of the model; the horizontal axis represents the number of clusters from 1 to 101 with a step of 5). Fig. S2. The further calibration of the Gaussian mixture models (the vertical axis represents the AIC value of the model; the horizontal axis represents the number of clusters from 20 to 60 with a step of 1). Fig. S3 Location and Spatial Boundaries of Toronto's Six Districts. [file 12942_2023_344_MOESM1_ESM.docx]

Additional file 1

We iterated the four covariance types and the number of clusters from 1 to 101 with a step of 5 to train the model and compared their results with the AIC values. Fig. A1 shows the initial calibration results, with different colours of lines representing the four different covariance types. In the figure, the minimum AIC is achieved using the "full" covariance with the number of clusters around 30 to 40. To find the best number of clusters, the models were further calibrated with the number of clusters set between 20 to 60 with a step of 1. As shown in Fig. A2, the best model performance (with the lowest AIC value) is achieved when the number of clusters equals 36.


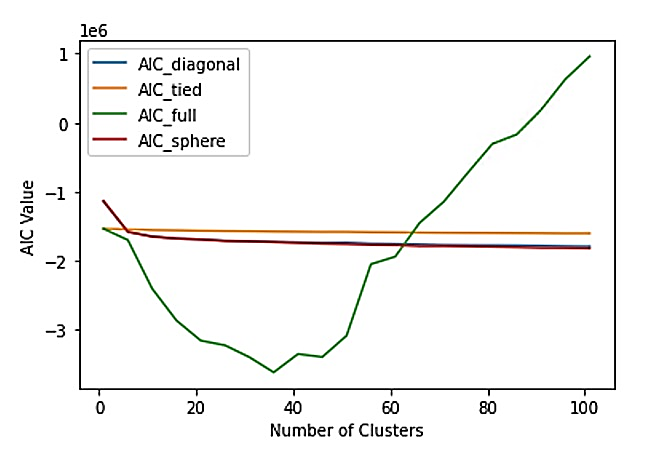


**Fig. S1** The initial calibration of the Gaussian mixture models (the vertical axis represents the AIC value of the model; the horizontal axis represents the number of clusters from 1 to 101 with a step of 5).


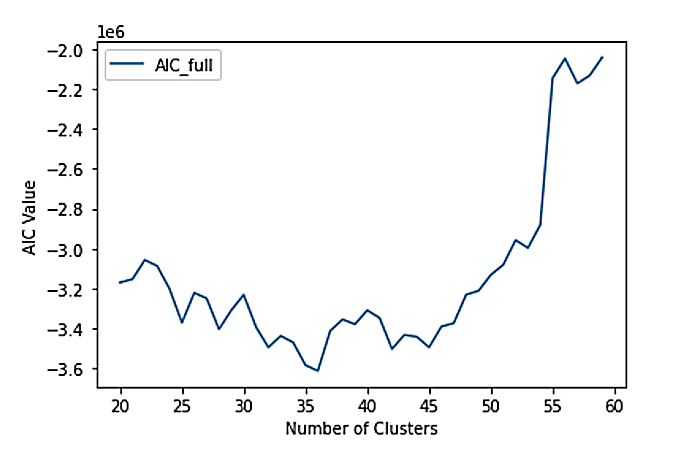


**Fig. S2** The further calibration of the Gaussian mixture models (the vertical axis represents the AIC value of the model; the horizontal axis represents the number of clusters from 20 to 60 with a step of 1).


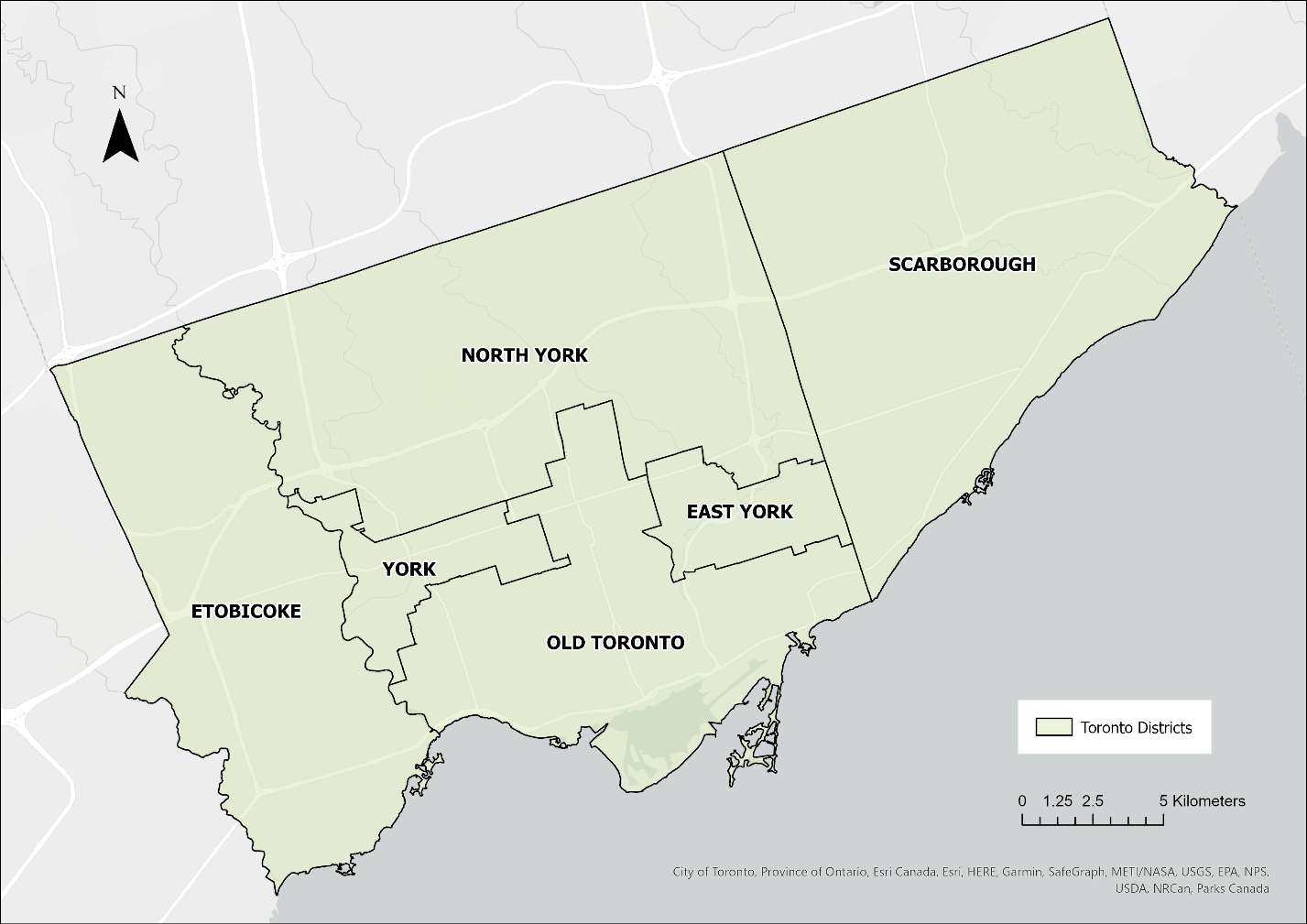


**Fig. S3** Location and Spatial Boundaries of Toronto's Six Districts.
